# The Role of Early Intervention Therapy in Neurodevelopmental Outcomes of Premature Infants

**DOI:** 10.7759/cureus.68618

**Published:** 2024-09-04

**Authors:** Sudhir Malwade, Shiji Chalipat, Priyanka Shah, Shailaja Mane

**Affiliations:** 1 Pediatrics, Dr. D. Y. Patil Medical College, Hospital and Research Centre, Dr. D. Y. Patil Vidyapeeth (Deemed to be University), Pune, IND; 2 Pediatric Neurology, Dr. D. Y. Patil Medical College, Hospital and Research Centre, Dr. D. Y. Patil Vidyapeeth (Deemed to be University), Pune, IND

**Keywords:** parental involvement, neurological outcomes, early intervention therapy, neonatal intensive care, prematurity

## Abstract

Neurodevelopmental impairments are known to be more common in premature infants. Premature and low birth weight babies are now more likely to survive because of modern technologies and advancements in perinatal and postnatal care. However, long stays and exposure to harsh stimuli in neonatal intensive care units are known to have a negative impact on the developing neonatal brain. Therefore, the goal of early intervention (EI) is to assist an infant and their family in achieving the greatest potential outcome. This encompasses a broad spectrum of strategies and structured programs that might differ in many aspects, such as who should implement them, where they should take place, and when they should begin. They aid in minimizing brain damage and optimizing growth via varied sensory and motor stimuli. Current evidence supports the initiation of EI therapy soon after birth, starting from neonatal intensive care units and continuing post-discharge. This research is important, especially in developing countries like ours, owing to the increasing number of premature deliveries due to multiple reasons. The focus of this article is to analyze the various protocols and applications available to us for the implementation of EI therapies and their benefits.

## Editorial

The proverb "prevention is better than cure" also holds true in this scenario, implying that it is preferable to act proactively rather than wait for problems to emerge. This is the basis of the early intervention (EI) ideology. The early years of life represent a critical window for brain development. This is a period of rapid growth, synapse formation, and neuronal plasticity, making the brain exceptionally receptive to environmental stimuli. Notably, interventions during this early critical period are expected to have a stronger impact since the developing brain exhibits plasticity [1]. Additionally, research suggests that babies born at lower gestation age appear to benefit best from intense EI [2].

EI capitalizes on this critical window by providing enriched environments, thus stimulating activities to foster brain development, sensory stimulation to enhance sensory processing and integration, motor skill development to promote gross and fine motor abilities, and language and cognitive stimulation. These domains can be achieved via varied approaches including visual/auditory stimulation and massage therapy with developmentally supportive care in the neonatal units. In addition to this, parental involvement is of vital importance. 

The bright and stimulating environment of a neonatal unit contrasts sharply with the dark, warm, and protective environment of the uterus, and therefore, exposure to such an environment during a critical time leads to both short-term and long-term outcomes, especially for premature infants. For term infants, some degree of parental separation and altered feeding patterns can also lead to adverse neurological outcomes. For all these reasons, it is imperative to begin developmentally supportive care and EI from the neonatal unit itself. This comprises touch stimulation in the form of kangaroo mother care - adequate skin-to-skin contact with the caregiver in an appropriate position. Children can have tactile experiences from being massaged, cuddled, rocked, fed, and changed in diapers [3]. These maneuvers form a sense of trust and establish a bond between the caregiver and the infant, thereby enhancing the self-esteem of the infant. The precise mechanism behind tactile stimulation is unclear. However, stimulation of insulin-like growth factor 1 (IGF-1) and insulin due to tactile stimulation helps in weight gain of infants. This, in turn, helps improve neuromotor behavior and muscle tone. In a randomized control trial conducted by Gianni et al., the relationship between early maternal touch and neurodevelopmental outcomes in infants with very low birth weight was studied [4]. The intervention group showed higher scores in personal, social, eye-hand coordination, and practical reasoning at 36 months. Another study conducted by Field et al. demonstrated the short-term advantages of tactile stimulation, such as healthy weight gain [5]. 

Early in fetal life, i.e., during the first 20 weeks of gestation, is the period of development of the fetal auditory system [3]. Ototoxic drugs and noise can harm the cochlea and affect the functions of the vestibular system. Excessive noise, such as that in the neonatal intensive care unit (NICU), can lead to hemodynamic instability in these neonates. Maximum noise levels in the NICU should not exceed 45 dB, according to the American Academy of Pediatrics (AAP) [6]. However, several times these guidelines are not met due to the varied functioning of each unit. This, in turn, leads to annoyance, sleep disturbances, and hypertension in these neonates. Sleep can be enhanced via introducing ‘quiet time’, during which a concentrated effort is made to reduce noise and disturbances for the infant. Along with this, music therapy has a role to play in the neurodevelopmental outcome of premature infants by enhancing synaptogenesis and cognitive development. This can be in the form of lullabies, nursery rhymes, and/or instrumental music. A randomized control trial conducted by Nelson et al. carried out auditory stimulation in preterm infants with severe central nervous system (CNS) injuries, and the outcome of these therapies showed better motor performance and 23% fewer cerebral palsy diagnoses at one year of age [7]. During the early developmental stage, synaptic pruning is enhanced by music therapy, which is essential for the brain's normal growth. The development of the neurological system, particularly the corpus callosum, is stimulated by repeated listening and music instruction.

Babies can be exposed to multiple adverse oral experiences in the form of suctioning, use of oro-gastric tubes, and/or long-term medications. These eventually can lead to feeding difficulties. There must be ways to improve the overall exposure to these experiences by using expressed breast milk on mouth-guards and the use of non-nutritive sucking to aid in the oro-motor skill development process [1]. 

Research has indicated that ambient light, as opposed to direct light, is crucial for healthy visual development [3]. According to AAP regulations, illumination levels for observation should be 650 lux, while for interventions, they must be no more than 2000 lux. Infants in neonatal units are also exposed to bright light during procedures as well as during phototherapy. This can damage the circadian rhythm and cause sleep disturbances in these neonates. Hence, by 32-34 weeks of corrected gestation, in order to establish a circadian rhythm, infants should be exposed to lighting that varies as per the diurnal variation. All measures must be taken to prevent infants from being exposed to direct light. Diurnally cycled light exposure improves weight gain and reduces the incidence of retinopathy of prematurity (ROP). Synaptic connections in the visual cortex can be stabilized by visual stimulation.

Other types of interventions include physiotherapy, occupational therapy, parent-infant relationship enhancement, infant stimulation, and developmental care. 

Supporting parents during this entire process is essential. Efficient access to psychological assistance from the neonatal unit and follow-up appointments as needed would be helpful. It has been shown that involving parents in the interventions helps the parent-infant bond to grow stronger. EI therapists and health visiting teams frequently employ the Brazelton Newborn Behavioral Observation (NBO) system, a unique relationship-building tool created to assist and maintain the parent-infant bond [1]. Preventative EI programs that are started in the first 12 months of life for preterm infants have the biggest effect on improving developmental outcomes. These programs are usually centered around sensitive and responsive parenting in addition to infant development.

Centers that use EI protocols were found to have a better neurodevelopmental outcome for infants on follow-up compared to centers that did not use them. Along with this, infants who received EI therapy turned out to have better motor and cognitive skills than infants who did not receive it. 

However, despite all its benefits, EI therapy has some limitations. A trained staff nurse/caretaker is required to carry out these therapies. These can be carried out by the parent as well post-discharge from the NICU, but again, this requires a high degree of motivation. Another requirement to carry out these EI therapies is that the neonate must be hemodynamically stable, which is a huge challenge considering that these neonates are born prematurely. 

For premature neonates, there are significant variations in the early intervention program (EIP) provided in the NICU and throughout the post-discharge period. It is not one thing in particular but a collection of interventions provided to a child and their family in order to maximize the neurodevelopmental outcome. Strong parental participation, along with accurate implementation of the various components of EI, is crucial. With the increasing amount of preterm deliveries, in a developing country like ours, cost-effective early intervention therapies ought to be standardized in our neonatal intensive care units for a better long-term outcome. 

## References

[1] Elliot-Smith A, Sammut A, Hutchon B, Merchant N, O’Brien F, Huertas-Ceballos A (2024). Early intervention to improve neurodevelopmental outcomes for high-risk infants. Paediatr Child Health.

[2] Anderson PJ, Treyvaud K, Spittle AJ (2020). Early developmental interventions for infants born very preterm - what works?. Semin Fetal Neonatal Med.

[3] Malwade SD, Chalipat SS, Karambelkar GR (2017). Role of early intervention therapy on long term neuro-developmental outcome of premature newborns. Indian J Basic Appl Med Res.

[4] Gianní ML, Picciolini O, Ravasi M, Gardon L, Vegni C, Fumagalli M, Mosca F (2006). The effects of an early developmental mother-child intervention program on neurodevelopment outcome in very low birth weight infants: a pilot study. Early Hum Dev.

[5] Field T, Diego M, Hernandez-Reif M (2010). Preterm infant massage therapy research: a review. Infant Behav Dev.

[6] Wachman EM, Lahav A (2011). The effects of noise on preterm infants in the NICU. Arch Dis Child Fetal Neonatal Ed.

[7] Nelson MN, White-Traut RC, Vasan U (2001). One-year outcome of auditory-tactile-visual-vestibular intervention in the neonatal intensive care unit: effects of severe prematurity and central nervous system injury. J Child Neurol.

